# Experimental study of the effects of nitroglycerin, botulinum toxin A, and clopidogrel on bipedicled superficial inferior epigastric artery flap survival

**DOI:** 10.1038/s41598-022-24898-9

**Published:** 2022-12-03

**Authors:** Mohamed A. Ellabban, Moustafa Elmasry, Islam Abdelrahman, Ghada Abdel Kader, Ingrid Steinvall, Folke Sjoberg, Amr A. Gomaa, Islam Omar Abdel Fattah

**Affiliations:** 1 Department of Hand Surgery, Plastic Surgery and Burns and Department of Biomedical and Clinical Sciences, Linköping University, 58183 Linköping, Sweden; 2 Plastic and Reconstructive Surgery Unit, Department of Surgery, Suez Canal University, Ismailia, 41522 Egypt; 3 Department of Human Anatomy and Embryology, Faculty of Medicine, Suez Canal University, Ismailia, 41522 Egypt

**Keywords:** Cell biology, Drug discovery, Biomarkers, Molecular medicine

## Abstract

Beneficial effects could be achieved by various agents such as nitroglycerin, botulinum toxin A (BoTA), and clopidogrel to improve skin flap ischaemia and venous congestion injuries. Eighty rats were subjected to either arterial ischaemia or venous congestion and applied to a bipedicled U-shaped superficial inferior epigastric artery (SIEA) flap with the administration of nitroglycerin, BoTA, or clopidogrel treatments. After 7 days, all rats were sacrificed for flap evaluation. Necrotic area percentage was significantly minimized in flaps treated with clopidogrel (24.49%) versus the ischemic flaps (34.78%); while nitroglycerin (19.22%) versus flaps with venous congestion (43.26%). With ischemia, light and electron microscopic assessments revealed that nitroglycerin produced degeneration of keratinocytes and disorganization of collagen fibers. At the same time, with clopidogrel administration, there was an improvement in the integrity of these structures. With venous congestion, nitroglycerin and BoTA treatments mitigated the epidermal and dermal injury; and clopidogrel caused coagulative necrosis. There was a significant increase in tissue gene expression and serum levels of vascular endothelial growth factor (VEGF) in ischemic flaps with BoTA and clopidogrel, nitroglycerin, and BoTA clopidogrel in flaps with venous congestion. With the 3 treatment agents, gene expression levels of tumor necrosis factor-α (TNF-α) were up-regulated in the flaps with ischemia and venous congestion. With all treatment modalities, its serum levels were significantly increased in flaps with venous congestion and significantly decreased in ischemic flaps. Our analyses suggest that the best treatment option for ischemic flaps is clopidogrel, while for flaps with venous congestion are nitroglycerin and BoTA.

## Introduction

Fasciocutaneous flaps are commonly used in the functional restoration of tissue defects after trauma and tumor resection^1^. However, complications such as flap ischemia and congestion leading to defective coverage are a possible risk^2^. In animal studies, the rat skin flap model has been used to identify flap survival factors and to test treatment protocols that could improve it. Random pattern flaps have been more frequently used than axial pattern flaps^3^.

In 2017, Matsomouto et al.^3^ introduced a new flap model of the reverse U-shaped abdominal fasciocutaneous flap based on the bilateral superficial inferior epigastric artery (SIEA) and vein (SIEV). Ligation of one of two SIEAs would produce a model of flap ischemia, and of the two SIEVs would produce a model of flap congestion. They showed consistent flap survival in the new model, which could be used to assess the effect of various agents on flap survival.

Previous studies have shown attempts to enhance the tolerance of the skin flaps to ischemia and congestion using different drug preparations^1,4^. Various agents have been stated to be beneficial in improving flap survival through inhibiting inflammation, getting rid of free radicals, improving angiogenesis, and/or increasing the microvascular network^1^. The ideal therapeutic agent should be effective and easily administrated, with low morbidity and cost-effectiveness^5^.

It is known that nitroglycerin ointment has a potent local vasodilator effect and increases the local blood flow that could potentially improve flap survival, although it is not popularly used for preventing skin flap necrosis, possibly due to the lack of effect shown in some studies^6,7^. However, others have shown desirable results on flap survival in both humans and animals^8–10^. Recent studies have shown a good effect of botulinum toxin A (BoTA) on skin and muscle flap survival. This effect is mainly due to vasodilatation and increased blood perfusion in the flap, especially when injected perivascularly^11–15^. Its vasodilator effect has been suggested via autonomic nerve block, thus increasing blood flow to the flap tissue ^16^.

Another medication with promising results is oral clopidogrel, a powerful antiplatelet agent which reduces platelet activation and aggregation. It has been shown that suppression of platelet aggregation could be an effective strategy in reducing flap necrosis. Additionally, it also has a vasodilatory effect^17^.

The aim of this study was to compare the effectiveness of nitroglycerin, BoTA, and clopidogrel on the reverse U-shaped bipedicled SIEA flap models of flap ischemia and congestion.

## Materials and methods

### Animals

In this experimental study, we included a total of 80 male Sprague–Dawley rats aged between 12 and 16 weeks with an average weight of 210 ± 30 g. The rats were grouped in separate cages, housed in a well-ventilated room with a 12 h dark/light cycle, and allowed tap water and rodent pellets ad libitum. Additionally, they were left for 2 weeks of adaptation before any procedure. The animals were handled gently and inspected daily for any discomfort or infection. All surgical procedures were done under complete anesthesia, and a postoperative course of analgesics and antibiotics was administered. In addition, all efforts were made to minimize animal suffering.

### Experimental design

The rats were randomly assigned to one of the two main groups, either the induced flap ischemia or the induced flap venous congestion group (40 rats in each group). Then each main group was further randomly subdivided into four subgroups (10 rats in each subgroup); a control group, a group treated with nitroglycerin ointment, a group treated with BoTA injection, and a group treated with oral clopidogrel.

### Surgical procedures

On the day of surgery, rats were anesthetized by an intraperitoneal injection of ketamine hydrochloride (Ketamax®, Troikaa, Gujarat, India) (50 mg/kg) and xylazine (Xylajet®, ADWIA, Egypt) (10 mg/kg)^18^. After confirming signs of deep anesthesia, the rats were put in a supine position. The hair over the abdomen was shaved, and then the skin was disinfected using a povidone-iodine solution wash.

**Figure 1:**
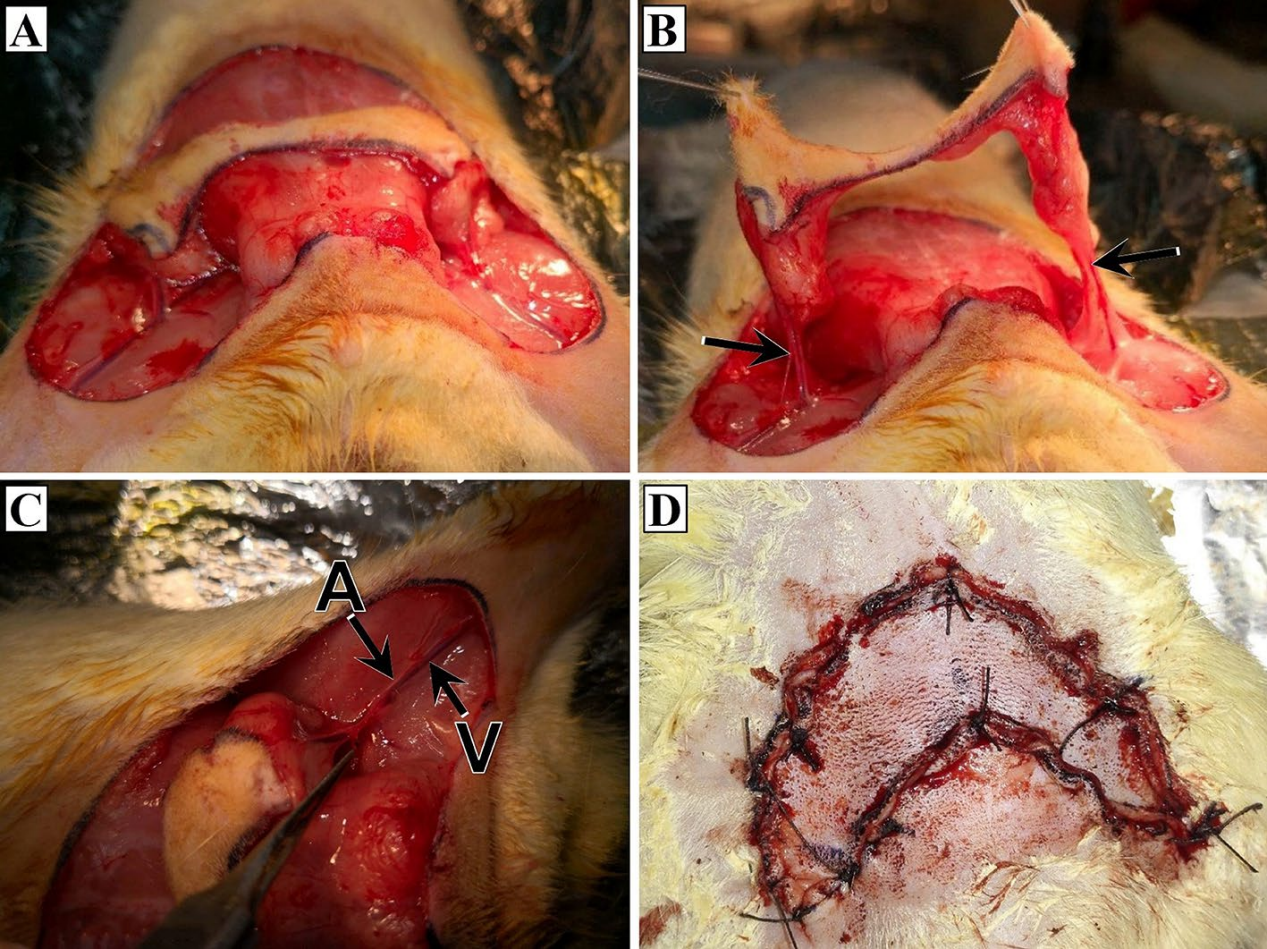
The surgical procedures. (**A**) The skin flap immediately after performing an incision. (**B**) Elevated skin with its panniculus carnosus preserving the supplying superficial inferior epigastric vessels at both sides (arrows). (**C**) The left superficial inferior epigastric artery (A) and vein (V) prior to ligation. (**D**) Repositioned skin flap after suturing with a nonabsorbable suture material.

A 1 cm wide reverse U-shaped transparent template was used to mark the outlines of the future dissected skin flap. The top of the reverse U would be located at the midline at midway between the xiphoid and pubis. Moreover, the two ends of the reverse U were presented at the pubis level. The skin with its panniculus carnosus was then elevated, preserving the supplying superficial inferior epigastric vessels at both sides (Fig. 1A–C). Both SIEA and SIEV on the right side were preserved as the pedicle. Under a dissecting microscope (Olympus®, Tokyo, Japan), the SIEA was dissected and ligated with nonabsorbable material, while the SIEV was left intact in the ischemia group. In the venous congestion group, the left SIEV only was dissected and ligated, while the SIEA was left intact. Finally, the flap was repositioned and sutured with 4–0 nonabsorbable suture materials (Fig. 1D)^3^.

### Administration of treatment agents

Nitroglycerin 2% ointment (Nitro-Bid®, Fougera, Altana Co., Melville, NY, USA) was locally applied directly over the skin of the flap area, forming a visible layer every 8 h from the day of operation until the time of sacrifice at day 7^19^. BoTA (Botox®, Allergan Inc., Irvine, CA, USA) 2 IU (1 ml) was once injected directly into the soft tissue around the left superficial inferior epigastric vessels, producing a swelling at the left pedicle of the flap^20^. Clopidogrel bisulfate (Plavix®, Sanofi Aventis, France) 75 mg tablets were crushed and dissolved in distilled water and given by oral gavage at a dose of 25 mg/kg/day starting immediately after the operation^21^.

### Flap survival assessment

The skin flaps of all rats were photographed by a digital camera (Nikon®, Tokyo, Japan) on the day of operation (day 1), day 3, and day 7. The photographs were assessed for visible signs of necrosis. The surface areas of necrotic and intact parts were calculated using ImageJ® software and expressed in cm^2^. The percentage of the necrotic areas in the flaps was calculated as affected skin area/total flap area × 100.

### Histopathological assessment

On day 7, all rats were euthanized by an overdose of ketamine and xylazine. For the pathological study, skin flaps were resected, and tissue specimens from the transitional zones between the healthy and necrotic areas were fixed using 4% paraformaldehyde. After embedding in paraffin blocks, 4 μm thick sections were stained with hematoxylin and eosin (H&E)^22^.

### Transmission electron microscope

Skin specimens of 1 mm^3^ in diameter were fixed for 2 h in 2.5% glutaraldehyde, followed by post-fixation with 1% osmium tetroxide for 1 h. Graded concentrations of alcohol were used to dehydrate the specimens and then they were embedded in resin. Semithin toluidine blue-stained sections were prepared to be examined under a light microscope (Olympus®, Tokyo, Japan) at a magnification of × 1000 to detect areas that were examined by the transmission electron microscope (TEM). Ultrathin sections were cut and double-stained with uranyl acetate and lead citrate for examination using a TEM (JOEL®, Columbia, South Carolina, USA) at the Electron Microscope Unit, Al-Azhar University, Cairo, Egypt^23^.

### Real-time reverse-transcriptase polymerase chain reaction

Real-time reverse-transcriptase polymerase chain reaction (RT-PCR) was used to assess the mRNA expression of vascular endothelial growth factor (VEGF) and tumor necrosis factor-α (TNF-α) in the flap tissue. Tissue samples were cryopreserved at − 80 °C till RT-PCR analysis. TRIZOL reagent (Shanghai Yusheng Biotechnology Co., Ltd., Shanghai, China) was used for RNA extraction from each biopsy specimen regarding the manufacturer's instructions. The expressions of VEGF and TNF-α were assessed using quantitative RT-PCR using an SYBR PrimeScript RT-PCR Kit (Invitrogen, Carlsbad, CA, USA) following the manufacturer's protocol using glyceraldehyde 3-phosphate dehydrogenase as an internal control. The used primers were as the following:

VEGF-165: forward, 5′-CGTCTACAGATGTGGGGGTTGC-3′; and reverse, 5′-ACTGGTTTGGGGCCTTGAGAG-3′.

TNF-α: forward, 5′-CAAGGCTCACAGTGATTTTCTGG-3′; and reverse, 5′-TCATACCAGGGCTTGAGCTCA-3′.

The relative mRNA expression was analyzed using the 2-ΔΔCT method^20^.

### Enzyme-Linked Immunosorbent Assay

Enzyme-Linked Immunosorbent Assay (ELISA) was used to evaluate serum VEGF and TNF-α levels. The rats fasted overnight, and the blood samples from the abdominal aorta were collected immediately after sacrifice and left until coagulation. The serum was separated by centrifugation at 3000 rpm for 15 min at 4 °C^24^. According to the manufacturer's instructions, VEGF and TNF-α specific kits (R&D Systems, MN, USA) were used to measure their levels.

### Statistical analysis

The statistical analysis was done with the statistical package for the social sciences (SPSS) version 24 (SPSS®, Chicago, USA). Quantitative values are presented as mean (95% CI). Analysis of variance (ANOVA) followed by Tukey's *post-hoc* test was used to compare the study groups. Probabilities of less than 0.05 were accepted as significant.

### Ethical statement

The authors declare that the study is reported in accordance with ARRIVE guidelines. The handling and care of the animals were conducted in compliance with the U.K. Animals (Scientific Procedures) Act, 1986 and associated guidelines, EU Directive 2010/63/EU for animal experiments. The protocols were approved by the Research Ethics Committee of the Faculty of Medicine, Suez Canal University (approval No. 4488 #).

## Results

### Flap necrotic area percentage

**Figure 2:**
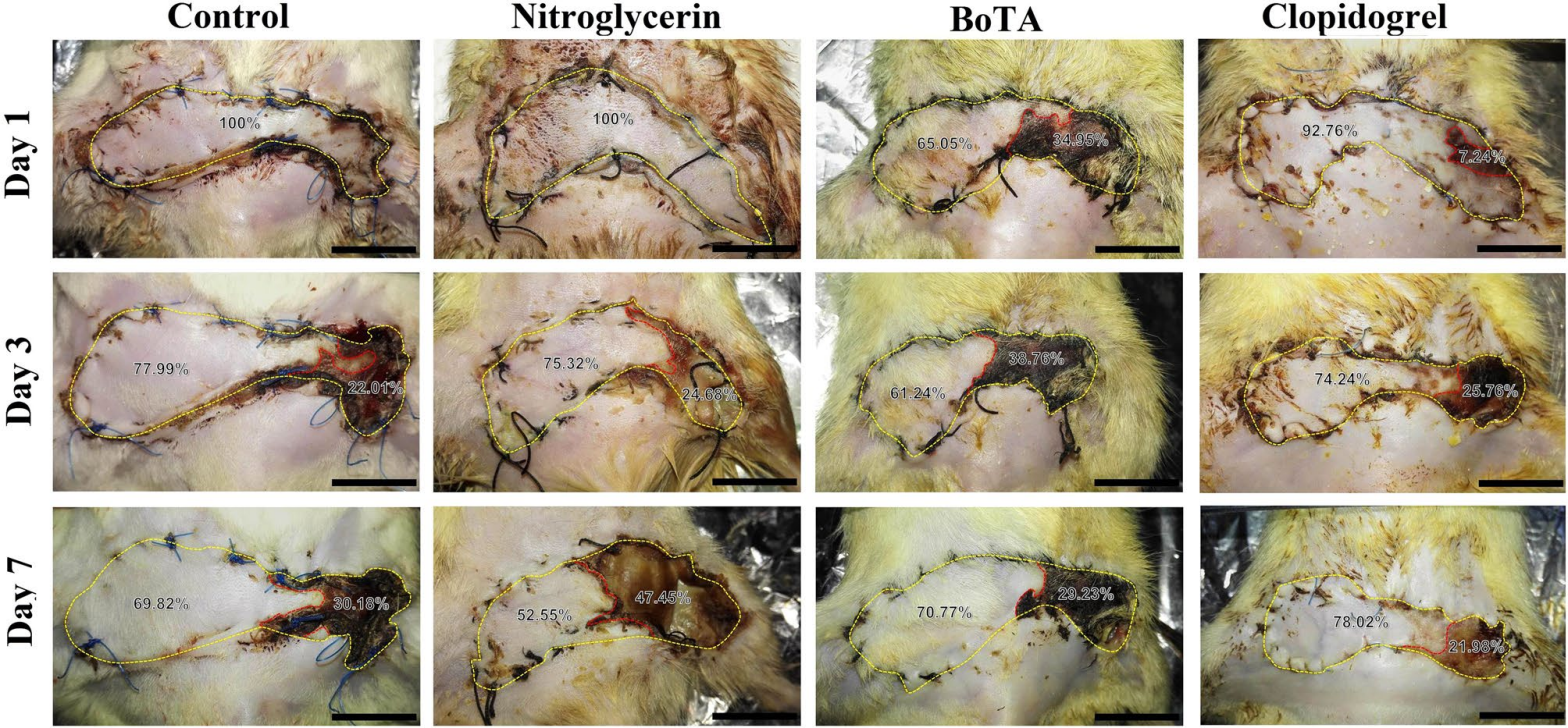
Gross appearance of skin flaps of the induced flap ischemia subgroups at days 1, 3, and 7 after the operation. The yellow dotted line outlines the total flap area and the demarcation between normal and necrotic tissues is indicated by the red dotted line. (Scale bar = 1.5 cm).

Among the induced flap ischemia subgroups, the clopidogrel subgroup showed a significant decrease in day 7 necrotic area percentage compared to the control, nitroglycerin, and BoTA subgroups (*p* < 0.001 for all). On the other hand, the necrotic area percentage was significantly increased in the nitroglycerin subgroup compared to the control on day 7 (*p* < 0.001) (Fig. 2 and Table 1).

**Table 1:** Different parameters of induced flap ischemia subgroups.

Parameters	Groups			
	Control	Nitroglycerin	BoTA	Clopidogrel
Necrotic area percentage	34.78 ± 4.56	53.47 ± 6.14^a^	39.02 ± 9.88^a,b^	24.49 ± 4.73^a,b,c^
VEGF_RT-PCR	1.11 ± 0.15	1.08 ± 0.05	3.74 ± 0.13^a,b^	3.55 ± 0.20^a,b^
TNF-α_RT-PCR	1.11 ± 0.12	1.20 ± 0.05	2.59 ± 0.28^a,b^	2.32 ± 0.09^a,b,c^
VEGF_ELISA	145.00 ± 1.41	167.83 ± 2.32^a^	290.17 ± 1.60^a,b^	215.00 ± 1.79^a,b,c^
TNF-α_ELISA	77.50 ± 1.05	68.00 ± 1.41^a^	49.33 ± 1.03^a,b^	59.00 ± 1.41^a,b,c^

Values are mean ± SD. ^a^*P* < 0.05 versus control group, ^b^*P* < 0.05 versus nitroglycerin group and ^c^*P* < 0.05 versus BoTA group. Statistical analysis was performed by one way ANOVA followed by Tukey's *post-hoc* test. *BoTA* botulinum toxin-A, *TNF-α* tumour necrosis factor-α, *VEGF* vascular endothelial growth factor.

**Figure 3:**
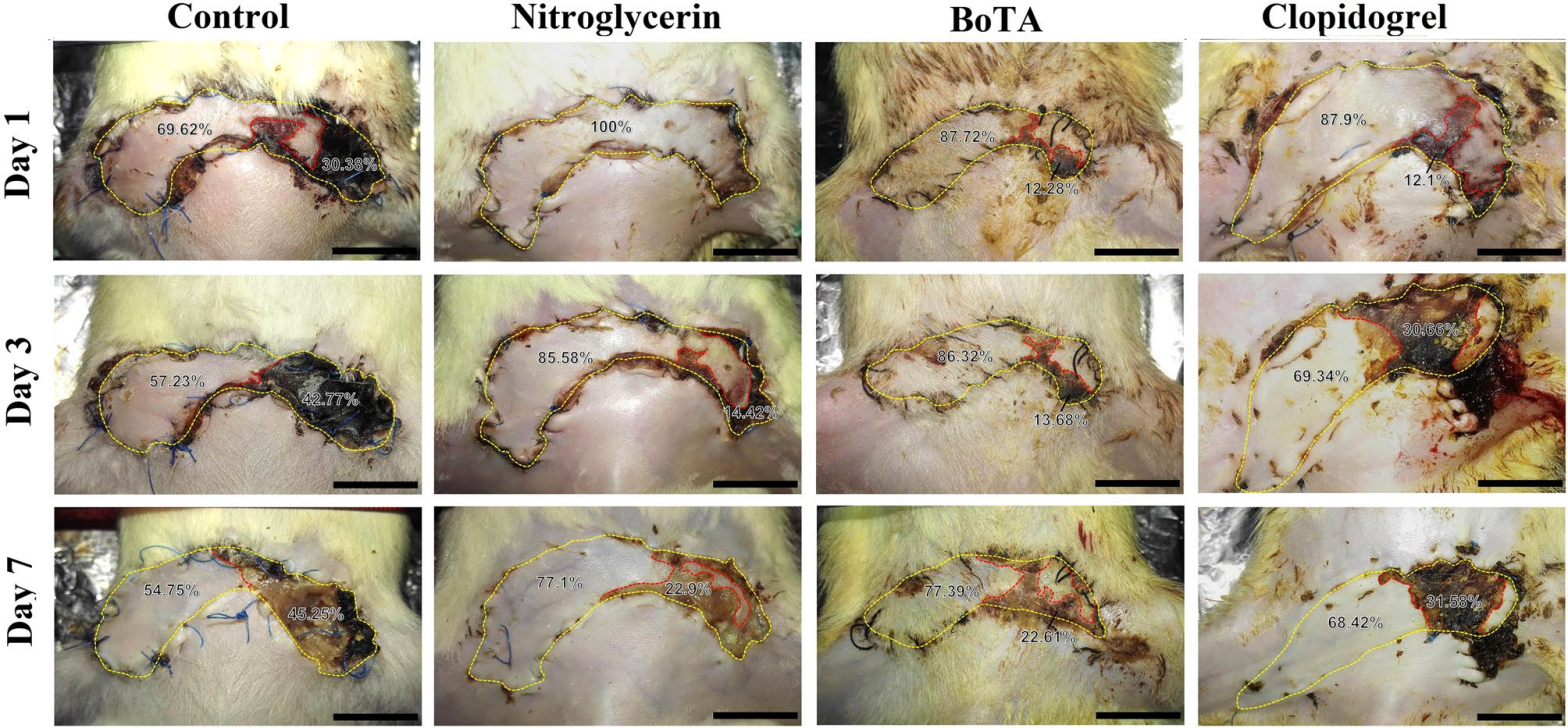
Gross appearance of skin flaps of the induced venous congestion subgroups at days 1, 3, and 7 after the operation. The yellow dotted line outlines the total flap area and the demarcation between normal and necrotic tissues is indicated by the red dotted line. (Scale bar = 1.5 cm).

Regarding the induced venous congestion subgroups, nitroglycerin, and BoTA treatment produced a significant decrease in the day 7 necrotic percentage compared to the control (*p* < 0.001 for all). Moreover, there was a significant decrease in the necrotic area percentage in the nitroglycerin subgroup compared to the BoTA and clopidogrel ones (*p* = 0.001 and *p* < 0.001, respectively). Nevertheless, there was no statistical difference between the control and clopidogrel subgroups (*p* = 0.301) (Fig. 3 and Table 2).

**Table 2:** Different parameters of induced flap venous congestion subgroups.

Parameters	Groups			
	Control	Nitroglycerin	BoTA	Clopidogrel
Necrotic area percentage	43.26 ± 3.55	19.22 ± 3.88^a^	28.92 ± 6.34^a,b^	39.18 ± 6.13^b,c^
VEGF_RT-PCR	0.96 ± 0.10	3.36 ± 0.32^a^	3.23 ± 0.12^a^	3.50 ± 0.18^a^
TNF-α_RT-PCR	0.99 ± 0.10	2.21 ± 0.12^a^	1.84 ± 0.16^a,b^	2.10 ± 0.03^a,c^
VEGF_ELISA	174.50 ± 2.59	276.83 ± 2.14^a^	243.00 ± 2.10^a,b^	245.00 ± 2.00^a,b^
TNF-α_ELISA	51.17 ± 2.04	92.17 ± 1.94^a^	95.00 ± 1.79^a^	88.17 ± 1.47^a,b,c^

Values are mean ± SD. ^a^*P* < 0.05 versus control group, ^b^*P* < 0.05 versus nitroglycerin group and ^c^*P* < 0.05 versus BoTA group. Statistical analysis was performed by one way ANOVA followed by Tukey's *post-hoc* test. *BoTA* botulinum toxin-A, *TNF-α* tumour necrosis factor-α, *VEGF* vascular endothelial growth factor.

### Histopathological findings

**Figure 4:**
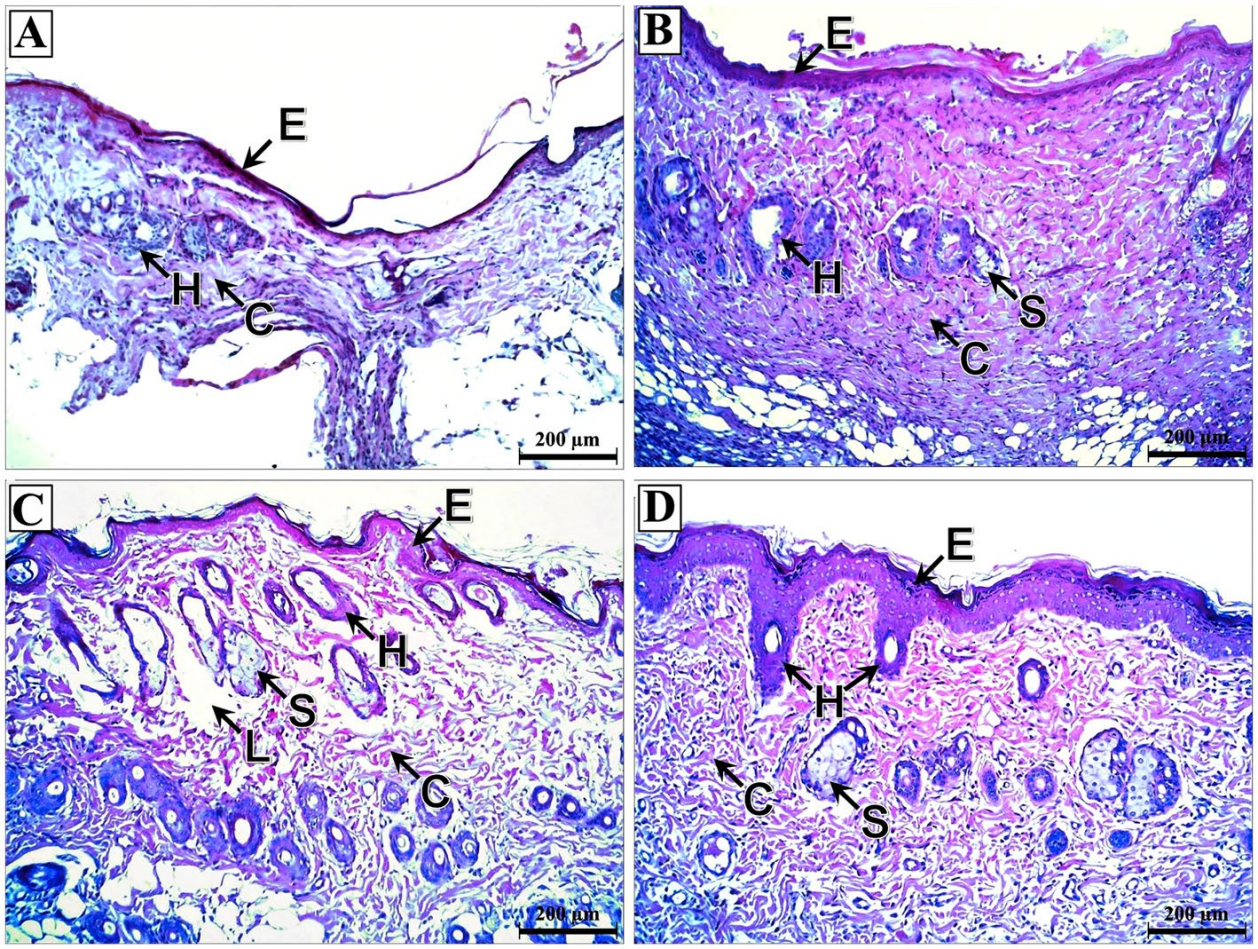
Photomicrographs of skin flap sections of the induced flap ischemia subgroups. (**A**) Control subgroup showing massive thinning of both epidermis (E) and dermis with loss of demarcations between the epidermal layers and extensive disorganization of the dermal collagen fibers (C). The hair follicles (H) are massively atrophied with the complete absence of sebaceous glands. (**B**) Nitroglycerin subgroup showing thin epidermis (E) with loss of the demarcations between its layers. The dermis reveals well-developed moderately disorganized collagen fibers (C) and massively atrophied hair follicles (H) and sebaceous glands (S). (**C**) BoTA subgroup showing relatively thin epidermis (E) and dermis with under-developed disorganized collagen fibers (C) having areas of their complete loss (L). The hair follicles (H) and sebaceous glands (S) are massively degenerated. (**D**) Clopidogrel subgroup showing restoration of the thickness of the epidermis (E) with distinct demarcations between its layers. There is a mild irregularity of the dermal collagen fibers (C), and well-developed hair follicles (H) and sebaceous glands (S). (H&E; × 100—Scale bar = 200 μm).

In skin flap sections of the induced flap ischemia subgroups, the control, nitroglycerin, and BoTA subgroups had thin epidermis and dermis with disorganized collagen fibers. They also had atrophied hair follicles and sebaceous glands (Fig. 4A–C). In contrast, clopidogrel subgroup skin flaps showed increased thickness of the epidermis and minimally irregular collagen fibers with well-developed hair follicles and sebaceous glands (Fig. 4D).

**Figure 5:**
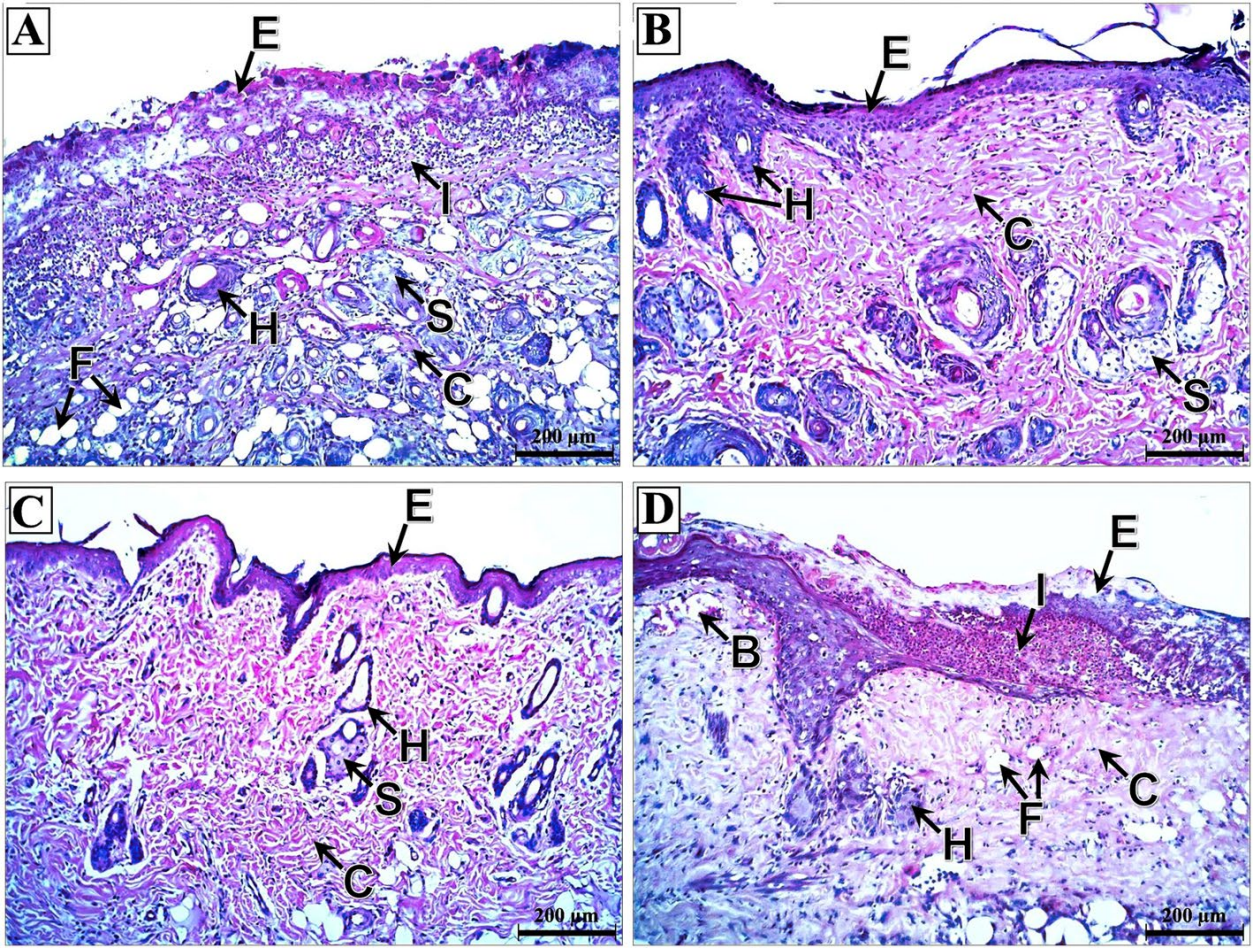
Photomicrographs of skin flap sections of the induced venous congestion subgroups. (**A**) Control subgroup showing massive necrosis of the epidermis (E) with mostly complete loss of its cells. The dermis reveals massive subepidermal inflammatory cell infiltration (I), extreme irregularity of collagen fibers (C), fatty infiltration (F) and severe degeneration of hair follicles (H) and sebaceous glands (S). (**B**) Nitroglycerin subgroup showing relatively thick epidermis (E), however, the stratum corneum is mostly lost. The dermis shows mostly regular collagen fibers (C) and well-developed hair follicles (H) and sebaceous glands (S). (**C**) BoTA subgroup showing relatively thick epidermis (E) with complete loss of the stratum corneum. The dermis demonstrates moderately irregular collagen fibers (C), massively degenerated hair follicles (H) and well-developed sebaceous glands (S). (**D**) Clopidogrel subgroup showing subepidermal inflammatory cell infiltration (I) with areas of massive coagulative necrosis of the epidermis (E). The dermis shows irregularity of its collagen fibers (C), fatty infiltrates (F), congested blood vessels (B), degenerated hair follicles (H) and a complete absence of the sebaceous glands. (H&E; × 100—Scale bar = 200 μm).

Regarding the induced venous congestion subgroups, there was extensive necrosis of the epidermis in the control subgroup with massive inflammatory cell infiltration, irregular collagen fibers, fatty infiltration, and degenerated hair follicles and sebaceous glands (Fig. 5A). However, the nitroglycerin and BoTA subgroup demonstrated restored epidermal thickness, regular collagen fibers and intact hair follicles and sebaceous glands (Fig. 5B,C). In the clopidogrel subgroup, there were areas of epidermal coagulative necrosis, while the dermis had irregular collagen fibers, inflammatory cellular infiltration, congested blood vessels, and degenerated hair follicles and sebaceous glands (Fig. 5D).

### Electron microscopic findings

**Figure 6:**
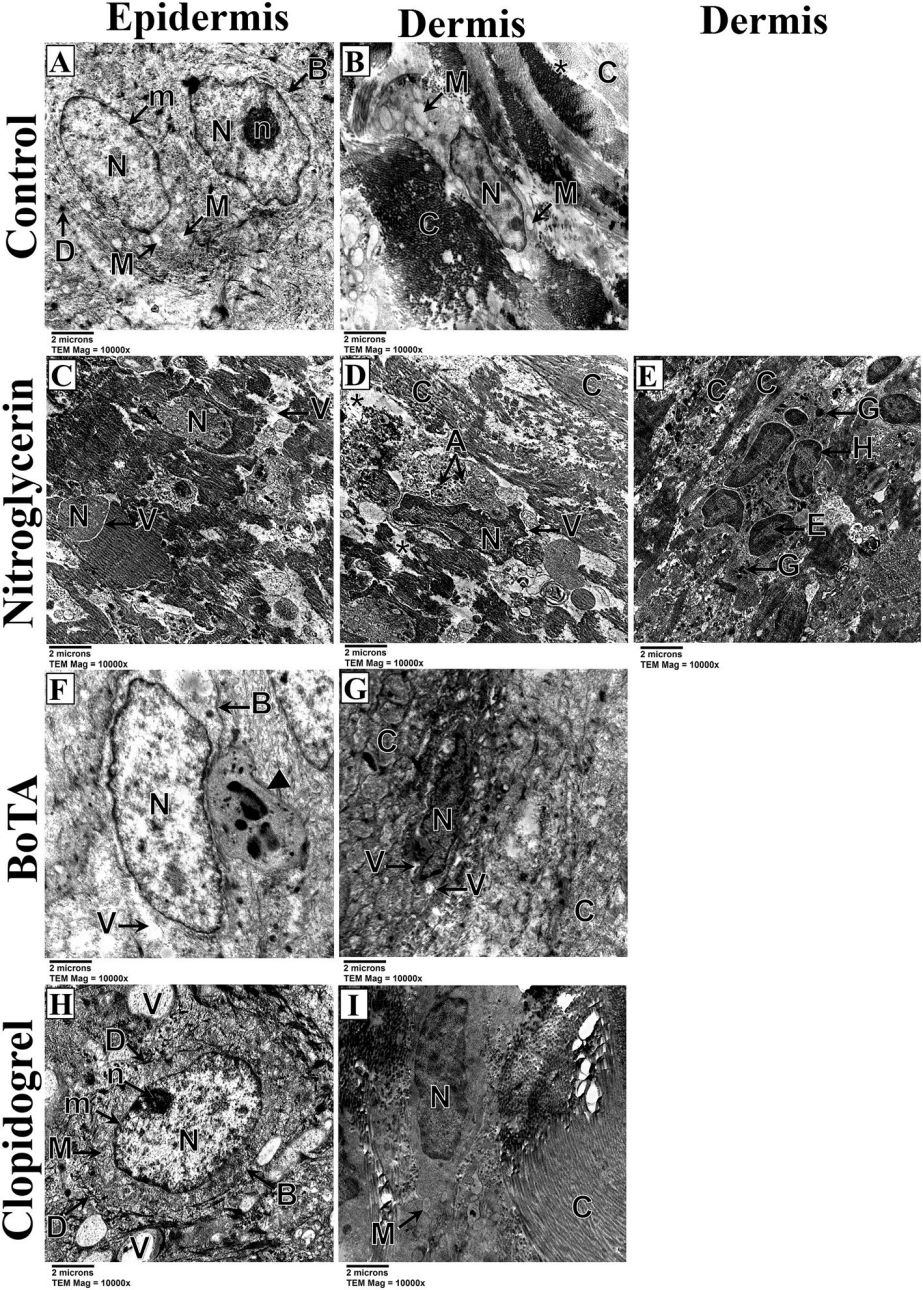
Electron micrographs of skin flaps of the induced flap ischemia subgroups; control (**A,B**), nitroglycerin (**C–E**), BoTA (**F,G**) and clopidogrel (**H,I**) subgroups. (**A**) The epidermis shows keratinocytes with euchromatic irregular nuclei (N), distinct nuclear membranes (m) and a distinct nucleolus (n) in one of them. The mitochondria are swollen with absent cristae (M). The cell boundaries (B) are distinct showing apparent desmosomes (D). (TEM; × 10,000—Scale bar = 2 µm). (**B**) The dermis shows a fibroblast with a relatively regular euchromatic nucleus (N) and edematous mitochondria with lost cristae (M). The collagen fibers are relatively regular and arranged in horizontal and vertical manners (C), however, there are areas of their absence (asterisk). (TEM; × 10,000—Scale bar = 2 µm). (**C**) The epidermis shows keratinocytes with massively degenerated nuclei that have homogenous euchromatin all over (N) and absent nuclear membranes. The cytoplasm shows loss of its organelles and massive vacuolations especially around the nuclei (V). The intercellular boundaries are mostly lost. (TEM; × 10,000—Scale bar = 2 µm). (**D**) The dermis shows a fibroblast with an irregular nucleus demonstrating peripheral chromatin condensation (N) and perinuclear vacuolation (V), and cytoplasmic apoptotic bodies (A). Note the presence of areas of highly packed collagen fibers (C), in addition to areas of their absence (asterisk). (TEM; × 10,000—Scale bar = 2 µm) € The dermis shows a neutrophil with a lobulated nucleus having marginal heterochromatin (H) and central bright euchromatin (E), and cytoplasmic granules (G). The surrounding collagen fibers show areas of condensation (C). (TEM; × 10,000—Scale bar = 2 µm). (**F**) The epidermis shows a keratinocyte having an euchromatic irregular nucleus (N). The cytoplasm is with lost organelles and cytoplasmic vacuolations (V). The cell boundaries are distinct (B), however, the desmosomes are absent. Note the presence of a neutrophil (arrowhead) beside the keratinocyte with a lobulated nucleus and cytoplasmic granules. (TEM; × 10,000—Scale bar = 2 µm). (**G**) The dermis shows a fibroblast with irregular cytoplasmic vacuolations (V) and irregular nucleus having peripheral chromatin condensation (N). Note the presence of highly irregular collagen fibers (C). (TEM; × 10,000—Scale bar = 2 µm). (**H**) The epidermis shows a keratinocyte with a regular oval nucleus (N), distinct nucleolus (n) and nuclear membrane (m), and normally appearing mitochondria with distinct cristae (M). The cell boundary (B) is highly distinct with well-developed desmosomes (D). Note the presence of extracellular vacuolations (V). (TEM; × 10,000—Scale bar = 2 µm). (**I**) The dermis showing a fibroblast with a regular euchromatic nucleus (N) and minimally swollen mitochondria with lost cristae (M). The surrounding collagen fibers are highly regular and parallel (C). (TEM; × 10,000—Scale bar = 2 µm).

Skin flaps of the induced flap ischemia showed keratinocytes having euchromatic irregular nuclei and swollen mitochondria with relatively regular collagen fibers that were absent in many areas in the control subgroup (Fig. 6A,B). Moreover, the nitroglycerin and BoTA subgroups demonstrated excessive degeneration of the keratinocytes that had degenerated nuclei, absent nuclear membranes, lost organelles and cytoplasmic vacuolations with areas of condensed collagen fibers (Fig. 6C–G). In contrast, keratinocytes of the clopidogrel subgroup had regular euchromatic nuclei, intact mitochondria, and distinct cell membranes and desmosomes, in addition to regular collagen fibers (Fig. 6H,I).

**Figure 7:**
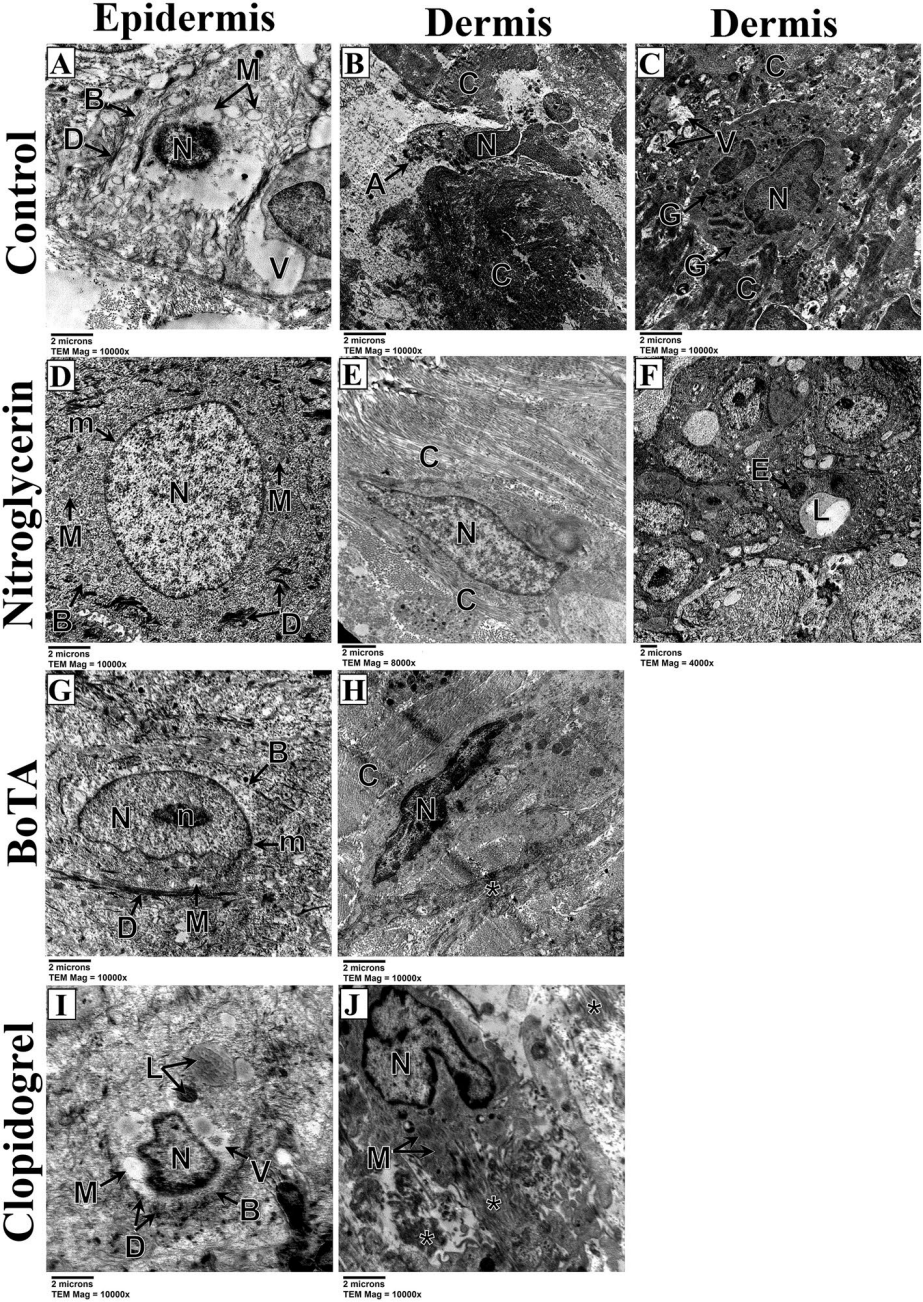
Electron micrographs of skin flaps of the induced venous congestion subgroups; control (**A–C**), nitroglycerin (**D–F**), BoTA (**G,H**) and clopidogrel (**I,J**) subgroups. (**A**) The epidermis shows a keratinocyte demonstrating degenerated nucleus with central condensation of its chromatin (N), swollen mitochondria with lost cristae (M) and cytoplasmic vacuolations (V). The cell boundaries are still distinct (B) with distinct desmosomes (D). (TEM; × 10,000—Scale bar = 2 µm). (**B**) The dermis shows a degenerated fibroblast having apoptotic bodies (A) and a nucleus with highly condensed chromatin (N). The surrounding collagen fibers are highly packed and irregularly arranged (C). (TEM; × 10,000—Scale bar = 2 µm). (**C**) The dermis shows a neutrophil with a lobulated nucleus (N), and cytoplasmic granules (G) and vacuolations (V) with surrounding irregular collagen fibers (C). (TEM; × 10,000—Scale bar = 2 µm). (**D**) The epidermis shows a keratinocyte with an euchromatic round nucleus (N) with a regular nuclear membrane (m). The cytoplasm shows intact mitochondria with preserved cristae (M). The cell boundary is distinct (B) with numerous desmosomes (D) (TEM; × 10,000—Scale bar = 2 µm). € The dermis shows a fibroblast with a flat regular euchromatic nucleus (N). The surrounding collagen fibers are mostly well-arranged (C) (TEM; × 8000—Scale bar = 2 µm). (**F**) The dermis shows a well-developed blood capillary having a lumen (L) and lined with an endothelial cell (E). (TEM; × 4000—Scale bar = 2 µm). (**G**) The epidermis shows a keratinocyte having a euchromatic nucleus (N) with distinct nucleolus (n) and a mostly regular nuclear membrane (m). The mitochondria are minimally swollen (M), however, their cristae are absent. The cell boundary is well-defined (B) with numerous desmosomes (D). (TEM; × 10,000—Scale bar = 2 µm). (**H**) The dermis showing a fibroblast with a mostly irregular nucleus having highly condensed chromatin (N). The surrounding collagen fibers show areas of well-arranged fibers (C) and other areas of poorly arranged ones (asterisk). (TEM; × 10,000—Scale bar = 2 µm). (**I**) The epidermis shows a highly degenerated keratinocyte with apoptotic shrunken irregular nucleus (N) and highly vacuolated cytoplasm (V). The mitochondria are highly swollen and vacuolated (M). There are lysosomes with different densities (L). Despite distinct cell boundary (B), desmosomes are highly minimized (D). (TEM; × 10,000—Scale bar = 2 µm). (**J**) The dermis shows a fibroblast with an irregular nucleus having peripherally condensed chromatin (N). The mitochondria are swollen with lost cristae (M). The surrounding collagen fibers are irregular and degenerated (asterisk). (TEM; × 10,000—Scale bar = 2 µm).

The control subgroup of the induced venous congestion revealed keratinocytes with degenerated condensed nuclei, swollen mitochondria, and cytoplasmic vacuolations; however, the cell boundaries and desmosomes were still distinct. In addition, the control subgroup showed irregular collagen fibers and neutrophilic inflammatory infiltration (Fig. 7A–C). Nevertheless, the nitroglycerin and BoTA subgroups had well-developed blood capillaries and regular collagen fibers, in addition to keratinocytes with euchromatic nuclei, intact mitochondria, and preserved desmosomes and cell membranes (Fig. 7D–H). The clopidogrel subgroup revealed degenerated collagen fibers and keratinocytes with shrunken nuclei, swollen mitochondria, lysosomes and cytoplasmic vacuolations (Fig. 7I,J).

### Real-time reverse-transcriptase polymerase chain reaction results

According to the induced flap ischemia subgroups, VEGF mRNA expression in the flap tissue was significantly higher than in the BoTA and clopidogrel subgroups compared to both control and nitroglycerin groups (*p* < 0.001), with no statistical difference between the BoTA and clopidogrel subgroups (*p* = 0.137) or between the control and nitroglycerin ones (*p* = 0.988). On the other hand, TNF-α mRNA expression was significantly increased in both BoTA and clopidogrel subgroups compared to the control and nitroglycerin ones (*p* < 0.001 for all), with a significant increase in the BoTA subgroup expression level compared to the clopidogrel one (*p* = 0.047). Nevertheless, there was no statistical difference between the control and nitroglycerin subgroups (*p* = 0.753) (Table 1).

The induced venous congestion subgroups demonstrated a significant increase in both VEGF and TNF-α mRNA expression levels in the 3 treated groups compared to the control (*p* < 0.001). However, there was no statistical difference between the 3 treated groups according to VGEF tissue expression. Furthermore, the nitroglycerin and clopidogrel subgroups showed a significant increase in TNF-α expression compared to the BoTA one (*p* < 0.001 and *p* = 0.003, respectively), with no statistical difference between both groups (*p* = 0.298) (Table 2).

### Enzyme-linked immunosorbent assay results

Regarding the induced flap ischemia subgroups, VEGF and TNF-α levels in the serum were significantly increased in the 3 treated subgroups compared to the control (*p* < 0.001), however, the highest levels of VGEF were in the BoTA subgroup in which VGEF level was significantly high compared to the nitroglycerin and clopidogrel ones (*p* < 0.001). On the other hand, the highest TNF-α level was in the clopidogrel subgroup, which was significantly higher than the other treated ones (*p* < 0.001) (Table 1).

According to the induced venous congestion subgroups, VEGF and TNF-α serum levels were significantly increased in the 3 treatment subgroups compared to the control (*p* < 0.001), with a significant increase in VEGF serum level in the nitroglycerin subgroup compared to the BoTA and clopidogrel ones (*p* < 0.001) with no statistical difference between both subgroups (*p* = 0.421). Nevertheless, the serum TNF-α levels in the nitroglycerin and BoTA subgroups were increased compared to the clopidogrel one (*p* = 0.006 and *p* < 0.001, respectively), with no statistical difference between the nitroglycerin and BoTA subgroups (*p* = 0.062) (Table 2).

## Discussion

The survival of the skin flap is determined mainly by its vascular pedicle patency^25,26^. So, skin flap surgery techniques were suggested to reduce the risks of arterial/venous occlusion and ischemia^25^. In addition, many therapeutic agents have been proven to enhance skin flap survival via the prevention of inflammation, providing free-radical scavenging, increasing angiogenesis, and/or enrichment of the microvascular network^27^.

To date, this is the first study comparing the effects of nitroglycerin, BTX‐A or clopidogrel treatments on 2 types of skin flap vascular insults, including arterial and venous insufficiency of the pedicled epigastric flaps in rats.

One of the most common complications of skin flap surgery is distal necrosis due to unpredicted thrombosis, vasospasm, and/or insufficient vascularity^28^. It is observed that venous thrombosis is more common and has a greater degree of irreversible damage than arterial thrombosis and, consequently, lower salvage rates^29^. Another flap- damaging factor is vasospasm which is a common complication in vascular pedicle flap surgeries causing temporary and incomplete obstruction of the vessels. However, it may be prolonged resulting in the formation of thrombi producing a complete obstruction of the vessels^30^. This vascular spasm may be attributed to the release of serotonin and certain prostaglandins from the platelets^31^. By the mechanisms discussed above, flap ischemia finally leads to reactive oxygen species accumulation, with consequent tissue damage^32^. The structural integrity of the skin is mainly induced by its collagen, and at the same time, fibroblasts are responsible for the secretion of its precursors: protocollagen types I and III^33^. As a cause, local flap hypoxia decreases collagen production and fibroblast replication, leading to impaired epithelialization^28^.

In the present study, the arterial and venous occlusions caused an increase in the percentages of the necrotic areas to 34.8 and 43.3%, respectively, by day 7. So, it was observed that a venous block has a more harmful effect than an arterial one. These results agreed with Matsumoto et al.^3^, who found that unilateral ligation of the SIEA resulted in necrosis of 38.2% of the abdominal flap surface area in correspondence to 57.7% in the SIEV blocked flaps by day 3, which was also confirmed by their histopathological results. Histopathological and ultrastructural examinations in our study also confirmed the hazardous effects of ischemia and congestion on the skin flaps revealing thinning of the epithelium, degradation of the dermal collagen, and degenerative changes concerning the keratinocytes and fibroblasts. Similar findings were reported in our previous study, which showed the effect of nicotine-induced ischemia on dorsal random pattern flap in rats^34^. The study showed that flap ischemia leads to keratinocytic degeneration, loss of desmosomes, mitochondrial damage, and the absence of blood vessels.

Nitroglycerin effectively causes vasodilatation by releasing nitric oxide leading to vascular smooth muscle relaxation and increased tissue perfusion with a main action on the veins, hence increasing the salvage response of the skin flaps^35,36^. Another known desirable effect is that nitroglycerin exerts an antithrombotic action^37^. Davis et al.^38^ reported that nitroglycerin ointment treatment with dorsal random pattern flaps decreased the percentage of flap necrosis from 44.1% in the control rats to 31.6% in nitroglycerin-treated rats and to 25.2% when nitroglycerin was combined with topical trolamine salicylate. In another study, Ghanbarzadeh et al.^39^ found that 2% nitroglycerin ointment decreased flap necrosis from 24%, in the control group, to 16.1%. However, we found that the most beneficial therapeutic effects of nitroglycerin are in the cases of venous congestion with little effect in the case of selective arterial ischemia with percentages of the necrotic surface area of 19.2% and 53.5%, respectively.

In the same context, we found that nitroglycerin restored the epidermal thickness, keratinocytic characteristics, and dermal collagen and fibroblast integrity only with venous-blocked flaps. On the other hand, nitroglycerin had a deleterious effect in the nitroglycerin arterial occlusion subgroup. Our previous work also reported that local nitroglycerin ointment has a significant salvage response in rats with dorsal random pattern flap nicotine-induced ischemia based on the histopathological and ultrastructural evaluation^34^. In addition, Aral et al.^40^ also reported that the topical application of NTG as a transdermal patch reduced the percentage of dorsal random pattern flap necrosis from 51.3 to 39.9% in rats.

In a guinea pig vascular model, it has been previously reported that BoTA attenuates the release of norepinephrine via SNAP-25 cleavage inducing vasodilatation^41^. In addition, BoTA induces the release of VEGF and can also increase nitric oxide synthase expression in skin flaps^13,42^. Furthermore, it improves wound closure, tissue remodelling and scar formation^43^.

In the present study, BoTA had better improvement effects in the venous congestion flaps than the ischemic ones, proved by histopathological and ultrastructural findings. In addition, the necrotic area percentages showed that BoTA worsens the salvage response of the flaps in arterial blocked flaps, which was 39% versus 34.8% in the control group. In another study conducted by Uchiyama et al.^13^ who reported that BoTA intradermal injection 24 h before the beginning of cutaneous ischemia–reperfusion injuries have protective effects by providing angiogenesis and prevention of hypoxia-induced tissue damage in mice. Ghanbarzadeh et al.^39^ also was injected BoTA intradermally but 2 weeks before dorsal random flap operation in rats. The necrotic area percentage significantly decreased from 56 to 24% versus the control rats. In addition to BoTA, the percentage was reduced to 16.1% when combined with the local application of nitroglycerin ointment. More specifically, Schweizer et al.^44^ produced a mouse model of dorsal flap arterial ischemia by dissection of the lateral thoracic artery. They injected BoTA subcutaneously near the vascular pedicle 24 h before or during the surgery. The blood flow of the flap was significantly increased, and, in contrast to our results, the necrotic surface area percentages declined from 38 to 16% when BoTA was injected preoperatively, and to 12% when injected intraoperatively when evaluated 5 days after surgery.

Clopidogrel is a thienopyridine derivative that inhibits platelet aggregation and is a prodrug that needs a hepatic cytochrome P450 enzyme termed P2C19 to change into its bioactive metabolites and inhibits platelet aggregation through irreversible blockade of P2Y12 (ADP) receptors on platelets^45^. Clopidogrel was given as a single preoperative oral dose in rats with McFarlane's skin flap operation by Akan et al.^46^. The necrotic surface area percentage was 53% in control rats and was reduced to 37% in clopidogrel-treated ones. Fatemi et al.^45^ also provided the rats with oral clopidogrel treatment but for 7 days postoperatively, which resulted in a reduction of the necrotic percentage from 38 to 28%. In comparison, the results of Vieira et al.^17^ showed a reduction of necrotic percentage from 51.61 to 27.70% when oral clopidogrel was administrated for 7 days postoperatively. In an epigastric flap model, rats were given clopidogrel for 7 days by Fichter et al.^47^. Clopidogrel in this research reduced the necrotic percentage from 81.13 to 36.47%. The results shown in the above publications were in accordance with our results, but only in the arterial occlusion group which showed a decrease in the necrotic percentage by 10.3%. However, the percentage was only reduced by 4.1% in the venous congestion one. Immediately after injury, the vascular smooth muscle cells produce a bulk of neointima increasing the vascular wall thickness^48^. However, clopidogrel is effective in inhibiting Angiotensin II‐induced vascular inflammation, intimal hyperplasia, and remodelling, by suppressing platelet activation and platelet‐monocyte binding^49^. At the same time, the blood flow is proportional to the cube of the radius of the vessel^50^. Thus, clopidogrel may indirectly increase arterial blood flow to the skin flap via increasing the arterial luminal caliber via decreasing the intimal thickness.

In the present study, VEGF tissue gene expression and serum level and TNF-α tissue mRNA expression were significantly increased in the treated arterial occlusion subgroups except in nitroglycerin one. However, the TNF-α serum levels were decreased in the 3 treated groups compared to the ischemic flaps. On the other side, VEGF and TNF-α tissue gene expressions and serum levels were significantly increased in the venous occlusion groups that received the 3 treatment modalities.

The VEGF signaling pathway is mainly involved in angiogenesis, which provides a vital role as a major therapeutic target for neovascular disorders, in addition to its role in the proliferation of endothelial cells, vasodilation, and increases vascular permeability^51,52^. Thus, increased VEGF levels in skin flaps are usually associated with an increase in skin flap survival^53^. On the other hand, TNF-α is produced by keratinocytes, vascular endothelial cells, and fibroblasts in response to injury, initiating the recruitment of inflammatory leukocytes into the injured tissues. In addition, TNF-α has a role in the regulation of fibroblast activity, keratinocytes, and endothelial cells and in the synthesis of extracellular matrix proteins and matrix metalloproteinases that are involved in the healing of the damaged tissues^54^. TNF-α is also known to stimulate the local release of angiogenic substances, such as basic fibroblast growth factor, VEGF, and platelet-derived growth factor subunit B, which triggers the proliferation and migration of endothelial cells and hence angiogenesis^55^. This may indicate that nitroglycerin, BoTA, and clopidogrel could increase flap survival by stimulating the angiogenesis and synthesis of the extracellular matrix that is essential in the healing process.

Our study revealed that flap ischemia and venous congestion have massive deleterious effects, including damage of the nuclei and mitochondria of the keratinocytes, while with flap ischemia, clopidogrel had an advantageous effect that was reflected in the minimal affection of the nuclei and mitochondria. On the other hand, with nitroglycerin treatment, the flaps with venous congestion were highly improved, showing preserved nuclei and mitochondria. However, with its treatment in the ischemic flaps, the nuclei were massively degenerated with lost organelles.

This research has some limitations. First, we were unable to use the same route of administration for the 3 treatment options, which might explain the difference in their curative response levels. Secondly, we could not use a direct measurement tool for the actual blood flow to the skin flaps in the different study groups as an important determinant factor for flap survival. Finally, we did not assess the time course of flap survival response to the treatment agents based on histopathological, VEGF and TNF-α gene expression, and blood level assessments.

Despite these limitations, our results provided evidence that the best treatment option for ischemic flaps was clopidogrel, while with BoTA and nitroglycerin treatments, necrotic flap area percentages were increased. On the other hand, flaps with venous congestion showed maximal mitigative responses to nitroglycerin, followed by BoTA, then clopidogrel.

## Conclusion

In conclusion, our results have emphasized that clopidogrel is effective in increasing salvage response, but only in skin flaps with ischemic insult, not in those with venous insufficiency; in which the nitroglycerin and BoTA are the ideal options for increasing survival rates.

## Data Availability

The datasets used and/or analyzed during the current study are available from the corresponding author on reasonable request.
